# Supplementary material for: MetaQTL: a package of new computational methods for the meta-analysis of QTL mapping experiments
Source: BMC Bioinformatics. 2007 Feb 8;8:49. doi: 10.1186/1471-2105-8-49 (PMC1808479; doi:10.1186/1471-2105-8-49)
Supplement: Additional File 7 — MetaQTL Package : jar file and tutorial. This Zip archive contains both the MetaQTL JAR file and the files of the tutorial. [file 1471-2105-8-49-S7.zip › org.inra.metaqtl/doc/org/metaqtl/bio/class-use/IBioEntity.html]

Uses of Interface org.metaqtl.bio.IBioEntity


|  |  |  |
| --- | --- | --- |
| **Overview** \| **Package** \| **Class** \| **Use** \| **Tree** \| **Deprecated** \| **Index** \| **Help** | |  |
| PREV   NEXT | **FRAMES**    **NO FRAMES**     **All Classes** |  |


---


## **Uses of Interface org.metaqtl.bio.IBioEntity**

| Packages that use IBioEntity | |
| --- | --- |
| **org.metaqtl.bio** |  |
| **org.metaqtl.bio.entity** |  |
| **org.metaqtl.bio.entity.adapter** |  |
| **org.metaqtl.bio.entity.factory** |  |

Uses of IBioEntity in org.metaqtl.bio

| Subinterfaces of IBioEntity in org.metaqtl.bio | |
| --- | --- |
| `interface` | `IBioAllele`             Class Description Here |
| `interface` | `IBioCross`             Class Description Here |
| `interface` | `IBioGenome` |
| `interface` | `IBioIndividual`             Class Description Here |
| `interface` | `IBioLGroup` |
| `interface` | `IBioLocus` |
| `interface` | `IBioOntology`             This interface defines some rules to handle a ontology. |
| `interface` | `IBioOntologyTerm`             This class defines a term of an ontology. |
| `interface` | `IBioPopulation`             Class Description Here |

| Methods in org.metaqtl.bio that return IBioEntity | |
| --- | --- |
| `IBioEntity` | `IBioEntity.getParent()`             Returns the entity that handles this entity. |
| `IBioEntity` | `IBioAdapter.toEntity(java.lang.Object obj)`             Convert the given bio entity object into another compliant object. |

| Methods in org.metaqtl.bio with parameters of type IBioEntity | |
| --- | --- |
| `java.lang.Object` | `IBioAdapter.fromEntity(IBioEntity obj)`             Convert the given compliant object to a bio entity object. |

Uses of IBioEntity in org.metaqtl.bio.entity

| Classes in org.metaqtl.bio.entity that implement IBioEntity | |
| --- | --- |
| `class` | `AlleleContainer`             Class Description Here |
| `class` | `BioEntity`             Class Description Here |
| `class` | `BioEntityCollection`             Class Description Here |
| `class` | `BioEntityContainer`             Class Description Here |
| `class` | `CrossPopulation`             Class Description Here |
| `class` | `GeneticMap`             Class Description Here |
| `class` | `GroupContainer`             Class Description Here |
| `class` | `Individual`             Class Description Here |
| `class` | `IndividualContainer`             Class Description Here |
| `class` | `LGroup`             Class Description Here |
| `class` | `Locus` |
| `class` | `LocusContainer`             Class Description Here |
| `class` | `Marker`             Class Description Here |
| `class` | `Ontology` |
| `class` | `OntologyTerm` |
| `class` | `Population`             Class Description Here |
| `class` | `QTL`             Class Description Here |

| Fields in org.metaqtl.bio.entity declared as IBioEntity | |
| --- | --- |
| `protected  IBioEntity` | `BioEntity.parent` |
| `protected  IBioEntity` | `BioEntityCollection.parent` |

| Methods in org.metaqtl.bio.entity that return IBioEntity | |
| --- | --- |
| `protected  IBioEntity[]` | `BioEntityContainer.entities()` |
| `protected  IBioEntity` | `BioEntityContainer.getEntity(java.lang.String name)` |
| `IBioEntity` | `BioEntity.getParent()` |
| `IBioEntity` | `BioEntityCollection.getParent()` |
| `static IBioEntity` | `BioEntity.newBioEntity(int type)` |

| Methods in org.metaqtl.bio.entity with parameters of type IBioEntity | |
| --- | --- |
| `protected  void` | `BioEntityContainer.addEntity(IBioEntity entity)` |
| `static Population` | `Population.newCrossPopulation(int type, java.lang.String name, IBioEntity parent)` |

Constructors in org.metaqtl.bio.entity with parameters of type IBioEntity

`AlleleContainer(java.lang.String name,
IBioEntity parent)`

`BioEntity(java.lang.String name,
IBioEntity parent)`

`BioEntityContainer(java.lang.String name,
IBioEntity parent)`

`CrossPopulation(java.lang.String name,
IBioEntity parent)`

`CrossPopulation(java.lang.String name,
IBioEntity parent,
int type)`

`GeneticMap(java.lang.String name,
IBioEntity parent)`

`GroupContainer(java.lang.String name,
IBioEntity parent)`

`Individual(java.lang.String name,
IBioEntity parent)`

`IndividualContainer(java.lang.String name,
IBioEntity parent)`

`LGroup(java.lang.String name,
IBioEntity parent)`

`Locus(java.lang.String name,
IBioEntity parent)`

`LocusContainer(java.lang.String name,
IBioEntity parent)`

`Marker(java.lang.String name,
IBioEntity parent)`

`Population(java.lang.String name,
IBioEntity parent)`

`QTL(java.lang.String name,
IBioEntity parent)`

Uses of IBioEntity in org.metaqtl.bio.entity.adapter

| Methods in org.metaqtl.bio.entity.adapter that return IBioEntity | |
| --- | --- |
| `IBioEntity` | `BioEntityCollectionAdapter.toEntity(java.lang.Object bean)` |
| `IBioEntity` | `GeneticMapBeanAdapter.toEntity(java.lang.Object bean)` |
| `IBioEntity` | `LGroupBeanAdapter.toEntity(java.lang.Object bean)` |
| `IBioEntity` | `LocusBeanAdapter.toEntity(java.lang.Object bean)` |
| `IBioEntity` | `OntologyBeanAdapter.toEntity(java.lang.Object obj)` |
| `IBioEntity` | `OntologyTermBeanAdapter.toEntity(java.lang.Object obj)` |

| Methods in org.metaqtl.bio.entity.adapter with parameters of type IBioEntity | |
| --- | --- |
| `java.lang.Object` | `BioEntityCollectionAdapter.fromEntity(IBioEntity obj)` |
| `java.lang.Object` | `GeneticMapBeanAdapter.fromEntity(IBioEntity obj)` |
| `java.lang.Object` | `LGroupBeanAdapter.fromEntity(IBioEntity obj)` |
| `java.lang.Object` | `LocusBeanAdapter.fromEntity(IBioEntity obj)` |
| `java.lang.Object` | `OntologyBeanAdapter.fromEntity(IBioEntity obj)` |
| `java.lang.Object` | `OntologyTermBeanAdapter.fromEntity(IBioEntity obj)` |
| `static void` | `BioEntityAdapter.fromEntity(IBioEntity entity, BioEntityBean bean)` |
| `static void` | `BioEntityAdapter.toEntity(BioEntityBean bean, IBioEntity entity)` |

Uses of IBioEntity in org.metaqtl.bio.entity.factory

| Methods in org.metaqtl.bio.entity.factory that return IBioEntity | |
| --- | --- |
| `abstract  IBioEntity` | `BioEntityFactory.load(java.io.InputStream stream)` |
| `IBioEntity` | `MapChartFactory.load(java.io.InputStream stream)`             TODO (non-Javadoc) |
| `IBioEntity` | `MapMakerFactory.load(java.io.InputStream stream)` |
| `IBioEntity` | `MapRawFactory.load(java.io.InputStream inputStream)` |
| `IBioEntity` | `MapTabFactory.load(java.io.InputStream stream)` |
| `IBioEntity` | `OntologyTabFactory.load(java.io.InputStream inputStream)` |
| `IBioEntity` | `XmlBioEntityFactory.load(java.io.InputStream stream)` |
| `abstract  IBioEntity` | `BioEntityFactory.load(java.io.Reader reader)` |
| `IBioEntity` | `MapChartFactory.load(java.io.Reader reader)`             TODO (non-Javadoc) |
| `IBioEntity` | `MapMakerFactory.load(java.io.Reader reader)` |
| `IBioEntity` | `MapRawFactory.load(java.io.Reader reader)` |
| `IBioEntity` | `MapTabFactory.load(java.io.Reader reader)` |
| `IBioEntity` | `OntologyTabFactory.load(java.io.Reader reader)`              Term\_id : the term identifient. |
| `IBioEntity` | `XmlBioEntityFactory.load(java.io.Reader reader)` |

| Methods in org.metaqtl.bio.entity.factory with parameters of type IBioEntity | |
| --- | --- |
| `abstract  void` | `BioEntityFactory.unload(IBioEntity entity, java.io.OutputStream stream)` |
| `void` | `MapChartFactory.unload(IBioEntity obj, java.io.OutputStream stream)`             TODO (non-Javadoc) |
| `void` | `MapMakerFactory.unload(IBioEntity entity, java.io.OutputStream stream)` |
| `void` | `MapRawFactory.unload(IBioEntity entity, java.io.OutputStream stream)` |
| `void` | `MapTabFactory.unload(IBioEntity entity, java.io.OutputStream stream)` |
| `void` | `OntologyTabFactory.unload(IBioEntity entity, java.io.OutputStream stream)` |
| `void` | `XmlBioEntityFactory.unload(IBioEntity obj, java.io.OutputStream stream)` |
| `abstract  void` | `BioEntityFactory.unload(IBioEntity entity, java.io.Writer writer)` |
| `void` | `MapChartFactory.unload(IBioEntity obj, java.io.Writer w)`             TODO (non-Javadoc) |
| `void` | `MapMakerFactory.unload(IBioEntity obj, java.io.Writer w)` |
| `void` | `MapRawFactory.unload(IBioEntity entity, java.io.Writer writer)` |
| `void` | `MapTabFactory.unload(IBioEntity obj, java.io.Writer writer)` |
| `void` | `OntologyTabFactory.unload(IBioEntity entity, java.io.Writer writer)` |
| `void` | `XmlBioEntityFactory.unload(IBioEntity obj, java.io.Writer writer)` |

---


|  |  |  |
| --- | --- | --- |
| **Overview** \| **Package** \| **Class** \| **Use** \| **Tree** \| **Deprecated** \| **Index** \| **Help** | |  |
| PREV   NEXT | **FRAMES**    **NO FRAMES**     **All Classes** |  |


---
